# Reporting of vertebral fragility fractures: can radiologists help reduce the number of hip fractures?

**DOI:** 10.1007/s11657-017-0363-y

**Published:** 2017-08-07

**Authors:** R. M. Mitchell, P. Jewell, M. K. Javaid, D. McKean, S. J. Ostlere

**Affiliations:** 1 Merton College, University of Oxford, Oxford, UK; 2 New College, University of Oxford, Oxford, UK; 3 Nuffield Department of Orthopaedics, Rheumatology and Musculoskeletal Sciences, University of Oxford, Oxford, UK; 4 Stoke Mandeville Hospital, Buckinghamshire Hospital NHS Trust, Aylesbury, UK; 5 Nuffield Orthopaedic Centre, Oxford University Hospital NHS Trust, Oxford, UK

**Keywords:** Vertebral fragility fracture, Hip fracture, Osteoporosis, Bone protection

## Abstract

**Summary:**

Patients with osteoporotic vertebral fractures are at increased risk of hip fracture. In a cohort of hip fracture patients, many had previous imaging studies showing incidental vertebral fractures. Fifty-four percent of fractures were not reported by the radiologist, highlighting a missed opportunity for diagnosing and treating osteoporosis, thereby preventing further fractures.

**Purpose:**

Patients with osteoporotic vertebral fragility fractures (VFFs) are at increased risk of future fractures, including hip fractures. Treating osteoporosis in these patients has the potential to reduce the risk of subsequent hip fractures, which are associated with high morbidity, mortality and cost. In this retrospective cohort study, we investigated the reporting and follow-up of VFFs evident on imaging by radiologists at the John Radcliffe Hospital, Oxford.

**Materials and methods:**

Data from the local Fracture Liaison Service was used to case-find all incident hip fractures from 2013 presenting to the trust. We then identified patients who had also undergone a radiological procedure that included the thoracic and/or lumbar spine in the previous 6 years. All identified radiological images were re-examined for the presence of VFFs using the Genant semi-quantitative method.

**Results:**

Seven hundred and thirty-two patients over the age of 50 with a hip fracture in 2013 were identified. One hundred and fifty-seven patients had previously undergone a radiological procedure involving the spine, and VFFs were identified in 65/157 (41%). Of these, only 30/65 (46%) were reported by a radiologist when the fracture was first visible. 32/35 (91%) of unreported VFFs were from imaging reported by non-musculoskeletal radiologists. Only 16/65 (25%) of patients with a VFF were documented as being on bone-specific therapy at the time of hip fracture.

**Conclusions:**

Our study highlights the under-reporting of osteoporotic vertebral fractures, particularly by non-musculoskeletal radiologists. Better systems for reporting and referring osteoporotic VFFs are necessary to increase the number of patients receiving appropriate osteoporosis treatment.

## Introduction

Osteoporosis is a systemic skeletal disorder characterised by low bone mass and microarchitectural deterioration resulting in an increased bone fragility and susceptibility to fracture [1]. Sufferers of osteoporosis are at an increased risk of fracture. One in 2 women and 1 in 5 men over the age of 50 will experience an osteoporotic fragility fracture in their lifetime [2]. Vertebral fragility fractures (VFFs) are the most common osteoporotic fracture [3]. VFFs can be identified from images performed specifically for examination of the spine (e.g. spinal radiographs, DXA vertebral fracture assessment) or incidentally (opportunistically) from images performed for other clinical indications (e.g. CT, MRI, lateral chest radiograph, abdominal radiograph) [4]. Despite being a common incidental finding on diagnostic imaging, the majority of fractures are asymptomatic [5]. However, it is well established that individuals with VFFs are at a significantly increased risk of suffering hip fractures and further vertebral fractures [6, 7]. Hip fractures are associated with increased morbidity and mortality, and in the UK account for at least £1.1 billion in hospital costs alone [8, 9].

VFFs seen on imaging are significantly under-diagnosed by radiologists [4], meaning a large proportion of patients are not receiving suitable, effective osteoporosis treatment. Reporting VFFs is an important component of the International Osteoporosis Foundation (IOF) Best Practice Framework for secondary fracture prevention [10, 11]. The recognised under-reporting of vertebral fractures stimulated the development of the Vertebral Fracture Initiative of the IOF [12], which provides an educational resource on the detection and management of these fractures for radiologists, clinicians and other healthcare professionals. Knowing that a patient has a vertebral fracture will also give more accurate assessments of future fracture risk using the WHO Fracture Risk Assessment Tool (FRAX) [13]. There are a wide range of treatments available for osteoporosis, which can reduce the incidence of future fractures by 30–65% [14]. Therefore, reporting of serendipitous VFFs by radiologists, and appropriate follow-up by the referring clinician, can help to reduce the number of hip fractures, and as a result, patient suffering and financial burden on the NHS.

In this retrospective cohort study, we investigated the prevalence and severity of osteoporotic vertebral fractures within a cohort of older patients who had recently suffered hip fractures. We then reviewed whether these vertebral fractures had been reported by the interpreting radiologist at the time of imaging.

## Methods

### Patient identification

A search was performed using our departmental radiology information database (RIS). This was used to identify all patients over the age of 50 who had suffered a hip fracture in 2013. From this cohort, we identified a subset of patients who also had imaging of the spine in the preceding 5 years within our NHS Trust, which included any CT (chest, abdomen, pelvis, colon or other), MRI (spine or other) or plain film (spine or lateral chest radiograph) in which at least five thoracic and/or lumbar vertebral bodies were clearly visible. Patients with the following conditions stated in the imaging request details or radiologist’s report were excluded from subsequent analysis: pubic rami fractures, peri-prosthetic fractures, history of high-velocity trauma, primary or metastatic bone malignancy, multiple myeloma or disorders of bone such as osteogenesis imperfect. Other exclusion criteria included unobtainable or poor quality images. Vertebral deformities were not recorded as fractured if judged to be secondary to Schmorl’s nodes or degenerative disc disease (spondylosis). Assessment of spinal imaging was also based on published normative values of anterior vertebral body vertical height to posterior vertebral body vertical height ratio to identify physiological vertebral wedging, most commonly seen at the thoracolumbar junction [15]. This study was registered as audit with our institution audit department.

### Identification of vertebral fractures

Imaging of the spine was assessed for the presence of vertebral fractures using the Genant semi-quantitative classification [16], classing fractures as grade 1 (20–25% height reduction), 2 (26–40% height reduction) or 3 (> 40% height reduction) depending on the extent of vertebral height loss by PJ and RM (medical students). All fractures identified using this method were reviewed by a consultant musculoskeletal radiologist (SO) to verify the presence of fractures, and to exclude vertebral deformity secondary to other factors. All reports from spinal imaging were evaluated for satisfactory reporting of any VFFs. Cumulative fracture score was calculated by the sum of the Genant fracture scores in a particular radiological image. Musculoskeletal radiologists were defined as those reporting imaging for musculoskeletal indications (X-ray spine or MRI spine).

### Treatment data

Treatment data was obtained from our institution’s Fracture Prevention Service database.

## Results

Seven hundred and thirty-two patients aged 50 years or above who had a hip fracture in 2013 were identified from our PACS database. Of these, 169 had X-ray, CT or MRI imaging of the spine on one or more occasions during the 5 years prior to their fracture. Twelve patients were excluded from subsequent analysis for the following reasons: unable to fetch/view scan (5), malignancy with bone metastases (4), osteogenesis imperfecta (1), bone lytic lesions (1) and high impact trauma (1). Of the remaining 157 patients, 66% were female, and mean age at the time of hip fracture was 82.5 years, with a range of 56 to 100 years.

### Reporting of vertebral fractures by radiologists

**Fig. 1:**
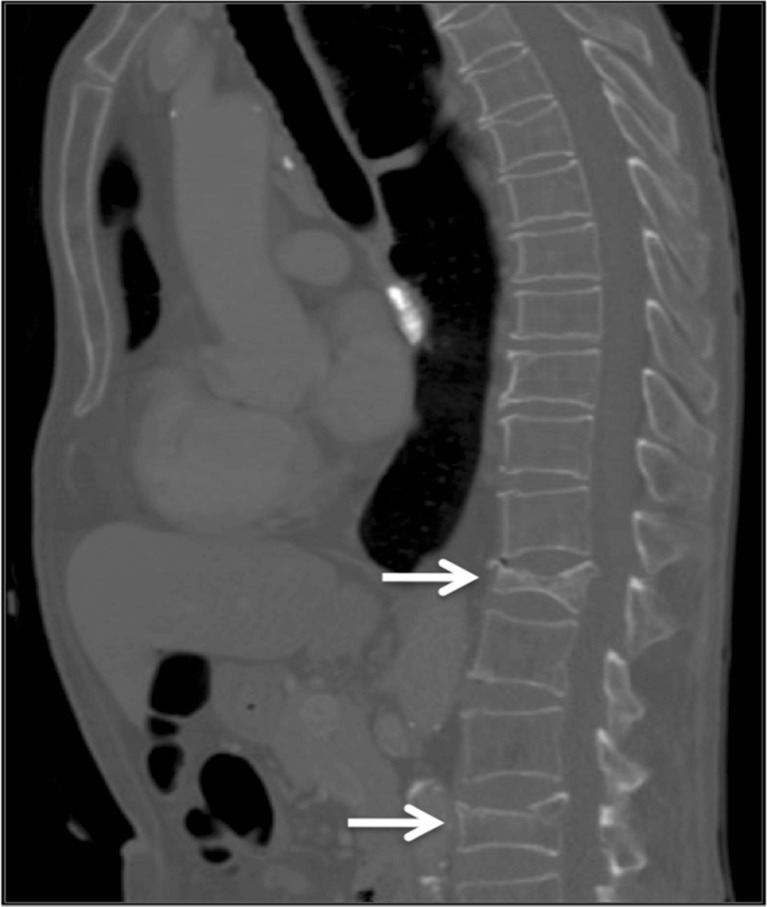
A sagittal section from a CT scan of a patient aged 71 years. The grade 3 L1 fracture and grade 2 L4 fracture (indicated) were not reported by the radiologist. The patient went on to suffer a hip fracture 3 years later

Of the 157 individuals who had spinal imaging prior to their hip fracture, 65 (41%) had detectable VFFs (Table 1). Only 30 (46%) of the VFFs first detectable on imaging were satisfactorily reported as ‘fractured’ by the interpreting radiologist at the time of imaging (Table 2), an example given in Fig. 1. Seven fractures were reported on a later date after additional spinal imaging. In imaging reported by musculoskeletal radiologists, 89% of fractures were reported, whereas in imaging reported by non-musculoskeletal radiologists only 14% of fractures were reported.

**Table 1:** Demographic of study cohort, with and without detectable VFFs

	No VFF detectable on imaging (*n* = 92)	VFF detectable on imaging (*n* = 65)
Hip fracture (*n* = 157)	92 (59%)	65 (41%)
Age (mean in years, range)	82 (56–94)	83 (62–100)
Gender (% female)	69%	63%

*VFF* vertebral fragility fracture

**Table 2:** Comparison of VFF reporting in hip fracture patients with VFF detectable on imaging

	Number of patients with detectable VFF on imaging (*n* = 65)	
	VFF reported at first opportunity (*n* = 30)	VFF not reported at first opportunity (*n* = 35)
Number of patients with detectable VFF on imaging (*n* = 65)	30 (46%)	35 (54%)
Age (mean in years; range)	84 (68–100)	83 (62–100)
Gender (% female)	67%	60%
VFF details		
Cumulative fracture score (mean; SD)	4.5 (3.20)	2.9 (2.23)
No. of fractures (mean; SD)	2.4 (1.65)	1.7 (1.05)
Imaging details (*n*, %)		
MRI spine^a^	7 (23%)	3 (9%)
MRI other	0 (0%)	0 (0%)
CT chest, abdomen, pelvis	2 (7%)	3 (9%)
CT chest ± abdomen^b^	2 (7%)	7 (20%)
CT abdomen ± pelvis	0 (0%)	3 (9%)
CT colon	1 (3%)	7 (20%)
CT other	0 (0%)	8 (23%)
X-ray spine^a^	18 (60%)	0 (0%)
Lateral chest X-ray	0 (0%)	4 (11%)

*VFF* vertebral fragility fracture, *SD* standard deviation

^a^Thoracic and/or lumbar spine

^b^Including CT pulmonary angiogram

**Fig. 2:**
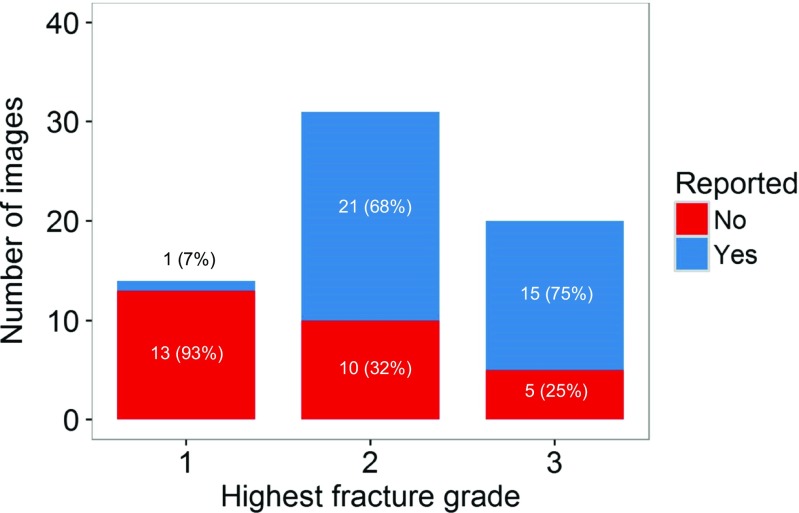
Number of images where the most severe grade of fracture was 1, 2 or 3. The *blue part* of each bar represents the number of images where a VFF was reported, and the *red part* represents images where the fracture was not reported

In images where the most severe grade of fracture was grade 1, only 7% of fractures were reported, compared with 68% of images where grade 2 was the most severe fracture, and 75% of images where grade 3 was the most severe (Fig. 2). The mean cumulative fracture score for VFFs on imaging where the fracture had been reported was 4.80, which was significantly higher than that for imaging where VFFs were not reported (mean = 2.91; *p* = 0.012). The number of vertebral fractures was also significantly higher on imaging where VFFs had been reported (mean = 2.40) compared with imaging where VFFs were not reported (mean = 1.69; *P* = 0.025).

### Bone-specific treatment in patients with vertebral fractures

Of those patients with a previously detectable VFF, only 16/65 (25%) were documented as taking bone-specific therapy at the time of hip fracture. Of these, 9 patients were taking alendronate, 3 risedronate and 4 calcium and vitamin D (data not shown). Of the 16 receiving therapy, a vertebral fracture had been reported in 13 patients (including 4 that were not reported at the first opportunity, but reported following later imaging). It was not possible to tell from our database whether treatment had been started because of a vertebral fracture being identified.

## Discussion

Our results reveal several important findings. Firstly, of the cohort of hip fracture patients, a significant proportion (over a fifth) had undergone previous imaging that included the spine. Of these patients, 41% had a detectable VFF. Taken together, these findings indicate a potential opportunity for detecting incidental vertebral fractures at an early stage.

Secondly, our data demonstrate that VFFs are significantly underreported by radiologists, particularly by non-musculoskeletal radiologists. Only 46% of visible vertebral fractures were recorded in the written report by the reporting radiologist at the time. However, the majority of fractures were recorded in reports by musculoskeletal radiologists. Non-musculoskeletal radiologists were less likely to identify vertebral fractures, particularly those of a lower Genant grade. On spinal X-rays, where the primary aim of imaging is to identify bony deformities, all fractures were reported. This suggests that it is not the detection of VFFs that presents a challenge to the radiologist, but lack of awareness of the need to look specifically for them. Vigilance for vertebral fractures should be increased in a population such as this where mean age is over 80 and therefore a high incidence of vertebral fractures should be anticipated [17].

Under-reporting of vertebral fractures by radiologists has been identified previously in several studies. Gehlbach et al. investigated the reporting of VFFs on lateral chest radiographs and found that only 52% of radiology reports contained notation of the presence of a vertebral fracture in the narrative of the report [18]. Vertebral fractures have also been shown to be under-reported on CT scans of the abdomen and/or pelvis [19] and MRI scans of the breast [20].

We also show that VFFs are more likely to be reported if there are multiple fractures, or if the fracture is more severe. This suggests that fractures causing obvious deformity to the spine are more likely to be commented on. However, in many cases, the spine may not be studied in sufficient detail to identify milder, less obvious fractures.

Only 25% of patients with vertebral fractures were reported to be receiving bone protection treatment at the time of their hip fracture. There are several reasons why patients with vertebral fractures may not go on to receive treatment for osteoporosis. Firstly, as we have demonstrated, vertebral fractures are often not reported by the radiologist, and at-risk patients are therefore not identified. Secondly, there may not be a robust system in place to ensure that patients who are discovered to have an osteoporotic fracture are referred for further follow-up and treatment. This is supported by a recent review of fracture liaison services globally which demonstrated that few hospitals have such a system of linking serendipitous vertebral fracture case finding for fracture liaison services [21]. Currently in our Trust, it is the responsibility of the general practitioner to follow up on the fracture and initiate further investigations and treatment. If the imaging was carried out for a complaint unrelated to the spine, treating the incidental finding of a vertebral fracture may not be prioritised or pursued. The county’s fracture liaison service is therefore currently unaware of a significant number of treatable cases of osteoporosis. To address this, we intend to introduce a system whereby patients with a vertebral fracture identified on a radiological report are directly referred to the local Fracture Prevention Service. A specialist fracture prevention nurse will automatically receive a list of all the vertebral fracture patients identified each month and will arrange follow-up investigations or treatment. Patients with a newly diagnosed VFF can then be easily identified from radiological reports and referred for appropriate follow-up and management.

Our study has some limitations. The retrospective nature of the study introduces an observer bias. We did not collect data on patients’ primary diagnosis or the indication for their imaging. We therefore cannot comment as to whether in studies with significant intra-thoracic or intra-abdominal findings, VFF were more likely to be overlooked. It is not always straight-forward differentiating fracture from deformity and the following factors are considered by the radiologist when making the distinction: presence of endplate abnormalities, loss of vertebral disc height and appearance in comparison to adjacent vertebrae. Even with a standardised method such as the Genant semi-quantitative technique, there is a room for subjectivity in the interpretation of imaging, particularly for grade 1 fractures. Finally, we had limited data on patients’ contraindications to osteoporosis medication. We therefore cannot comment on the number of cases where bone protection could not be commenced as a result of contraindications to therapy such as drug hypersensitivity, mechanical problems of the oesophagus (such as oesophageal stricture, dysmotility or achalasia) or those with severe renal dysfunction. Finally, we could not identify cross sectional imaging performed by hospitals outside the catchment of our NHS Trust.

## Conclusions

A significant proportion of patients with osteoporotic hip fractures have had previous spinal imaging in which VFFs are detectable. VFFs are more likely to be reported if there are multiple fractures, if the fracture is more severe or if reported by a musculoskeletal radiologist. However, less than half of detectable VFFs are reported at the time of imaging by the interpreting radiologist, representing consistent under-diagnosis of these fractures. This is a missed opportunity for the identification of patients at risk of further fractures. It is essential that radiologists have a high degree of vigilance for the presence of VFFs on routine imaging, particularly in older patients, to allow appropriate follow-up and initiation of effective osteoporosis treatment. This may have a significant impact in reducing the number of subsequent hip fractures and the associated morbidity, mortality and financial burden.
